# Sentiment analysis and topic modeling for COVID-19 vaccine discussions

**DOI:** 10.1007/s11280-022-01029-y

**Published:** 2022-02-25

**Authors:** Hui Yin, Xiangyu Song, Shuiqiao Yang, Jianxin Li

**Affiliations:** 1 School of IT, Deakin University, Geelong, Australia; 2 School of Computer Science and Engineering, University of New South Wales, Sydney, Australia

**Keywords:** COVID-19 vaccine, Sentiment analysis, Topic modeling, Data visualization

## Abstract

The outbreak of the novel coronavirus disease (COVID-19) has been ongoing for almost two years and has had an unprecedented impact on the daily lives of people around the world. More recently, the emergence of the Delta variant of COVID-19 has once again put the world at risk. Fortunately, many countries and companies have developed vaccines for the coronavirus. As of 23 August 2021, more than 20 vaccines have been approved by the World Health Organization (WHO), bringing light to people besieged by the pandemic. The global rollout of the COVID-19 vaccine has sparked much discussion on social media platforms, such as the effectiveness and safety of the vaccine. However, there has not been much systematic analysis of public opinion on the COVID-19 vaccine. In this study, we conduct an in-depth analysis of the discussions related to the COVID-19 vaccine on Twitter. We analyze the hot topics discussed by people and the corresponding emotional polarity from the perspective of countries and vaccine brands. The results show that most people trust the effectiveness of vaccines and are willing to get vaccinated. In contrast, negative tweets tended to be associated with news reports of post-vaccination deaths, vaccine shortages, and post-injection side effects. Overall, this study uses popular Natural Language Processing (NLP) technologies to mine people’s opinions on the COVID-19 vaccine on social media and objectively analyze and visualize them. Our findings can improve the readability of the confusing information on social media platforms and provide effective data support for the government and policy makers.

## Introduction

The outbreak of the novel coronavirus disease (COVID-19) has caused immeasurable losses to the world and affected the normal lives of billions of people around the world. COVID-19 is an infectious disease caused by the SARS-CoV-2 virus. Its common symptoms include cough, shortness of breath, fever, sore throat, and loss of taste or smell.^1^ Most people infected with the virus will experience mild to moderate respiratory illness and recover without requiring special treatment. However, some will become seriously ill and require medical attention. Older people and those with underlying medical conditions like cardiovascular disease, diabetes, chronic respiratory disease, or cancer are more likely to develop serious illness. Anyone can get sick with COVID-19 and become seriously ill or die at any age.^2^ According to the latest news from the WHO, as of August 23, 2021, more than 200 million people have been infected, and over 4.42 million people have died of the COVID-19^3^. It is considered one of the most severe epidemics of this century, comparable to past pandemics such as the Spanish flu in 1918 and the Black Death in the mid-13th century [28]. Eliminating COVID-19 has become a common goal of the world. Many countries and regions have adopted a series of specific measures to help slow down the spread of COVID-19. Such as closing borders, reducing the activities in public places (e.g., restaurants, gyms, shopping centers), working/studying from home, restricting travel distance and maintaining good hygiene. These measures have achieved remarkable results in controlling the spread of the epidemic, and some restrictive measures in some countries and regions have been gradually lifted. However, the recent COVID-19 Delta variant is more contagious, and countries worldwide have fallen into a state of emergency again. It seems that the existence of COVID-19 will last for a long time in the future. Based on history experience, vaccinations are the only long-term solution to this pandemic, provided that most of the population gets injections. Therefore, since the outbreak of the pandemic, countries and companies worldwide have initiated vaccine development and clinical trials.

As of August 23, 2021, there are 139 vaccine candidates and 22 of them have been approved in different countries across the world^4^. The Pfizer/BioNTech vaccine was the first to receive emergency validation by the World Health Organization (WHO) on December 31, 2020^5^, followed by AstraZeneca, Covishield, Janssen, Moderna. Subsequently, each country approved some vaccines and formulated specific policies to encourage all citizens to get vaccinated. According to statistics on August 23, 2021, 31.7% of the world population have received at least one dose of a COVID-19 vaccine, and 23.7% are fully vaccinated^6^. The vaccination rate (fully and partly vaccinated) against COVID-19 is 59.47% in USA, 72.69% in Canada, and 69.73% in England. In contrast, some countries have very low vaccination rates, such as 31.06% in India, 9.22% in Iran, 42.29% in Mexico, and 18.09% in Pakistan. The current vaccination rate has not yet reached the minimum requirements for controlling the spread of the pandemic in various countries. Excluding the reasons for the shortage of vaccines, there are other reasons that lead to the low vaccination rate. One possible reason is that people do not know enough about vaccines and are skeptical about their safety. They worry that the vaccine may cause long-term chronic diseases because the vaccine has not been adequately tested. Another possible reason is that the spread of false information about COVID-19 on social media may encourage those who hesitate or doubt the vaccine to oppose it. Therefore, by analyzing the discussions on social media platforms, early detection of people’s attitudes towards vaccines and timely response are conducive to the promotion of vaccines. For example, if people have more discussions about side effects after vaccination on social media platforms, more publicity can be carried out to inform the symptoms of side effects, how to relieve them, and how to deal with critical situations. People’s concerns have received a rapid response from the government, showing that the government has a fully understanding of vaccines, which can help increase people’s confidence and promote vaccination. Therefore, it is necessary to figure out people’s concerns about vaccines in the promotion of vaccines.

**Fig. 1:**
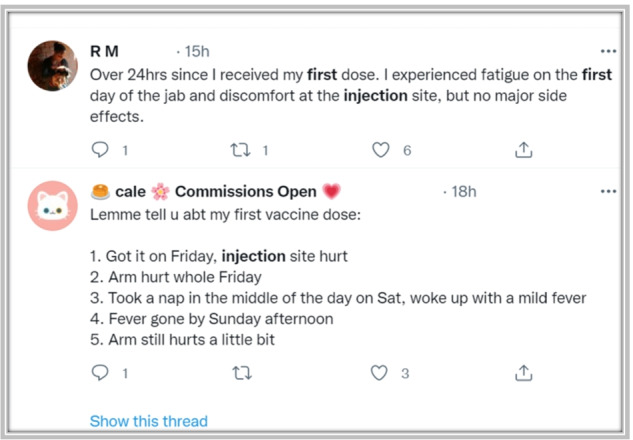
Two example tweets related to COVID-19 vaccine

Social media platforms (e.g., Twitter, Facebook, Instagram) and online forums (e.g., StackOverflow, Kaggle, Yahoo) provide a convenient way for communication. People can freely post, comment, express their opinions on specific topics or communicate with others on these platforms [9, 21]. Therefore, the discussions of the COVID-19 vaccine on social media provide us with a source of data to find out people’s concerns about the vaccine. Figure 1 shows two tweets related to COVID-19 vaccine on Twitter. Social media data is also a widely used source of information by other researchers[1, 11, 15, 20, 25].

This paper examines the discussions about the COVID-19 vaccine on Twitter, extracts the topics and the sentiment polarity in the tweets. Rather than looking at the worldwide, this paper examines four countries with the highest number of tweets during the study period. These tweets dominate the direction of public opinion and usually represent the majority of people’s opinions. In addition, analyzing people’s attitudes towards different vaccines, then performing visualization can help the government understand the people’s conditions and take corresponding measures when necessary.

The highlights for this work are summarized as follows:

- To the best of our knowledge, this is the first analysis of the public discussions related to the COVID-19 vaccines on social media since the emergence of the COVID-19 Delta variant.
- We adopted two robust text mining techniques: Latent Dirichlet Allocation (LDA) and Valence Aware Dictionary and Sentiment Reasoner (VADER), to extract the hidden information buried in noisy social media discussions.
- We conducted a comprehensive analysis of COVID-19 related discussions on Twitter. We found that discussions are mostly positive, and the dominant sentiment of trust means a high acceptance of the COVID-19 vaccine.

The paper is organized as follows. We review the related work in Section 2. Section 3 introduces the data preprocessing process and makes an in-depth exploration of the dataset. The methods used in this study are detailed in Section 4. Section 5 presents the process of data analysis and visualizes the results. Conclusions are made in Section 6.

## Related work

Social media such as Twitter, Facebook and Weibo have been widely used as data sources for analysing health-related problems [5, 14]. The outbreak of the COVID-19 pandemic has led to millions of discussions or posts on social media every day. Such a large number of user-generated posts provide a valuable source of data and thus receive great attention from researchers [18, 19, 23, 26]. Lots of research works have been carried out on social media to analyse and mine helpful information for analysing this pandemic.

### Sentiment analysis for COVID-19 based on social media

Some work exploited sentiment analysis as a tool to investigate people’s reactions during the pandemic through their posts on social media. Li et al. [12] analyzed the posts of Americans and Chinese on Twitter and Weibo during the pandemic from January 20, 2020 to May 11, 2020. They compared the emotions (i.e., anger, disgust, fear, happiness, sadness, surprise) and the emotional triggers (e.g., what a user is angry/sad about) to reveal sharp differences in The results showed that most people were confident in controlling the pandemic, but people’s sentiments such as fear, sadness and disgust were also appeared around the world. Zhou et al. [27] extracted five months of COVID-19 related tweets from Twitter to analyze the sentiment dynamics of people living in the state of New South Wales (NSW), Australia during the pandemic period. They divided tweets according to the level of local government areas (LGA) and observed the dynamic changes in sentiment over time. Yin et al. [24] proposed a novel framework to dynamically analyze the topic and sentiment of 13 million tweets related to COVID-19. They found that the proportion of positive tweets was slightly higher than negative tweets during the study period (2 weeks), which was consistent with other similar work. This work further analyzed the daily hot topics about the COVID-19 pandemic and found the common concerns discussed by people during the study period. For example, staying at home to ensure safety, the latest case reports, and people dying from the pandemic.

### Infodemic analysis for COVID-19 based on social media

In addition to being a valuable data source, social media has been described as a source of toxic “infodemic” (i.e., information of questionable quality). During the COVID-19 pandemic, vast infodemics have been generated worldwide mixed with false/fake or misleading information in the digital and physical environment. It causes confusion and risk-taking behaviors that can harm health, leads to mistrust in health authorities and undermines public health response^7^. Some work has focused on this type of information on social media during the pandemic period. Yang et al. [22] comprehensively studied the spread of prevalent myths related to COVID-19 people’s participation with them, and people’s subjective feelings about myths. They found that myths about the spread of infection and preventive measures spread faster than other myths, such as “5g corona is truth”, “Eating garlic can prevent the COVID-19”. People were most worried about the spread of coronavirus, and the common emotion among people was fear. Gallotti et al. [7] noticed that infodemic spread rapidly and widely through social media platforms during the pandemic. This information may mislead the public or increase social panic. Therefore, while the government and the people were fighting against the COVID-19 virus, they must also fight against infodemic. They analyzed more than 100 million Twitter messages posted worldwide during the early stages of the epidemic and then classified the reliability of the news being circulated. Furthermore, an Infodemic Risk Index was developed to capture the magnitude of exposure to unreliable news across countries. To contribute to the fight against the infodemic, Bang et al. [2] aimed to achieve a robust model for the COVID-19 fake-news detection task proposed in CONSTRAINT 2021 (FakeNews-19). They further improved the robustness of the model by evaluating different COVID-19 misinformation test sets (Tweets-19) to further improve the generalization ability of the model to solve the COVID-19 fake news problem in online social media platforms.

### Analysis of COVID-19 vaccine discussions on social media

With the development and promotion of vaccines, many researchers have carried out research work on COVID-19 vaccine related discussions on social media. Kwok et al. [10] extracted topics and sentiments related to the COVID-19 vaccine from Australian Twitter users between January and October 2020. They employed R library package *syuzhet* to score each tweet into two sentiments (positive, negative) and eight emotions (anger, fear, anticipation, trust, surprise, sadness, joy, and disgust). They found that two-thirds of all tweets expressed positive opinions and one-third expressed negative opinions. Finally, they identified three LDA topics in the dataset: (1) attitudes toward COVID-19 and its vaccination, (2) advocacy of infection control measures against COVID-19, and (3) misconceptions and complaints about COVID-19 control. Lyu et al. [13] used the same methods as [10] to identify sentiments and topics over a long time span in public discussions related to the COVID-19 vaccine on social media, with the goal of better understanding public perceptions, concerns and emotions that may influence the achievement of herd immunity goals. For the topic modeling, they yielded 16 topics, which were grouped into five overarching themes. Bonnevie et al. [4] quantified the increase in Twitter conversations around vaccine opposition during the COVID-19 pandemic in USA. They first collected such tweets, classified them into topics, and then tracked them. After four months of observation, they found a noticeable increase in vaccine opposition on Twitter. Exposure to this growing opposition to vaccines may mislead people against vaccines, which may significantly impact population health in the coming decades. Therefore, to ensure the widest support for a COVID-19 vaccine, it is essential to identify and address the messages used by vaccine opponents. Thelwall et al. [17] conducted a study to understand what types of vaccine hesitancy information shared on Twitter might be helpful in designing interventions to address misleading attitudes. The main themes discussed were conspiracies, vaccine development speed, and vaccine safety. The majority (79%) of those who refused vaccines on Twitter expressed right-wing views, fear of the deep state, or conspiracy theories. A significant proportion of those who refused vaccination (18%) tweeted about other topics in a mainly apolitical manner.

## Data preprocessing and statistics

For this study, we focus on analyzing the topics and sentiments of the COVID-19 vaccine related discussions on Twitter. As of August 23, 2021, there are 139 vaccine candidates, and 22 of which have been approved by different countries, and 192 countries with approved vaccines. For example, vaccines such as Pfizer, Oxford/AstraZeneca and Sinovac have been approved in USA, England and India, respectively. We adopt the latest publicly available dataset of the COVID-19 vaccine tweets from Kaggle^8^. The period for the collected data is from December 12, 2020 to July 2, 2021, and the dataset covers seven popular vaccine brands^9^ shown in Table 1.

**Table 1:** The COVID-19 vaccine brands in the dataset

Vaccine Brand	Description
Pfizer/BioNTech	Approved in 97 countries, 27 trials in 15 countries.
Sinopharm	Approved in 60 countries, 9 trials in 7 countries.
Sinovac	Approved in 39 countries, 19 trials in 7 countries.
Oxford/AstraZeneca	Approved in 121 countries, 39 trials in 20 countries.
Moderna	Approved in 69 countries, 25 trials in 6 countries.
Covaxin	Approved in 9 countries, 7 trials in 1 countries.
Sputnik V	Approved in 71 countries, 20 trials in 7 countries.

We preprocessed the original dataset by the following steps. Firstly, as the location of a tweet is necessary information in this study, we first deleted the tweets without location information and got 78,827 tweets. After that, we removed the noisy words from the remaining tweets. The procedures include: (1) Removing the Twitter handles, URLs, emojis, and hashtags; (2) Removing non-English words or common words that do not provide insights into a specific topic (e.g., stop words); (3) Case folding (i.e., lowering the case of words to allow for lexical processing); (4) Lemmatization to remove inflected endings and return a word to its base or dictionary form. (5) Investigating the combination of two words (bigrams) to ensure that words such as “side_effect” could be one token instead of separating “side” and “effect”. We also removed tweets with a length less than four words after processing, which usually cannot provide reasonable semantics. In the end, we got 75,665 tweets for our experimental study.

**Fig. 2:**
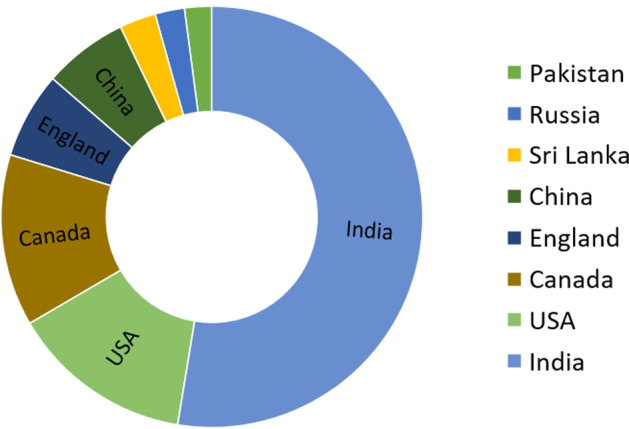
The distribution of tweets of top eight countries in the dataset

Figure 2 shows the top eight countries with the largest number of tweets and their proportions. India accounts for more than half of the posts, which is 52.59%. Such a high volume of tweets may be related to the out-of-control pandemic in India, the shortage of vaccines^10^ and a large number of users. People are more actively participating in the discussions of the COVID-19 vaccine.

According to the COVID-19 vaccine official website^11^, in Table 2, we list the approved vaccines for the four countries with the most tweets in the dataset. The four countries are India, USA, Canada and England. In fact, the vaccines approved in each country are not limited to these seven brands, but we only study the most popular seven vaccines in this study.

**Table 2:** Statistics on COVID-19 vaccines approved in four countries

Vaccine Brand	India	USA	Canada	England
Pfizer/BioNTech		$\surd$	$\surd$	$\surd$
Sinopharm				
Sinovac				
Oxford/AstraZeneca	$\surd$		$\surd$	$\surd$
Moderna	$\surd$	$\surd$	$\surd$	$\surd$
Covaxin	$\surd$			
Sputnik V	$\surd$			

We only count the seven brands in this study. In fact, each country has approved more vaccines

**Fig. 3:**
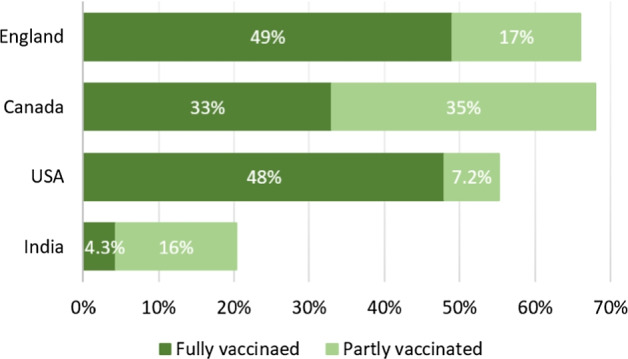
Share of people vaccinated against COVID-19, as of July 2, 2021

Figure 3 shows the share of people vaccinated against COVID-19 in the four countries as of July 2, 2021 (end of data collection). The vaccination dataset uses the most recent official numbers from governments and health ministries worldwide. Population estimates for per-capita metrics are based on the United Nations World Population Prospects.^12^ Obviously, in England, Canada, and USA, the proportion of people vaccinated is much higher than in India, whether fully or partially vaccinated. Based on the above statistics, we have come to the conclusion that India’s vaccination rate is the lowest among the four countries, but the proportion of discussions on Twitter is the largest.

## Adopted methods for topic modeling and sentiment analysis

We employ two methods for in-depth analysis of the COVID-19 vaccine discussions on Twitter. The first one is Valence Aware Dictionary for sEntiment Reasoning (VADER) for sentiment analysis, and the second one is Latent Dirichlet Allocation (LDA) for topic modeling.

### Measuring tweet sentiment

Sentiment analysis (SA), also known as opinion mining, aims to automatically mine the opinions, attitudes, and feelings in texts, has a wide range of applications. We use VADER [8] to analyze the sentiment polarity of tweets in this study. VADER is a lexicon and rule-based sentiment analysis tool specifically attuned for sentiments expressed in social media. Its sentiment lexicon includes all lexical features of existing well-established & human-validated sentiment lexicons (LIWC, ANEW, GI) and common expressions in social media text, such as emoticons, acronyms, slang. Besides the sentiment polarity of the lexicon, it also contains sentiment intensity information. More than 9,000 token features were rated on a scale from “[-4] Extremely Negative” to “[+4] Extremely Positive”, “[0] represents Neutral (or Neither, N/A)”. They retain the lexical features of all non-zero mean scores and had a standard deviation of less than 2.5 determined by the sum of these ten independent raters. This leaves over 7,500 lexical features with validated value scores indicating the polarity of the emotion (positive/negative), and the intensity of the emotion from -4 to +4. VADER uses the compound score as the final sentiment score of a sentence, which is very effective when dealing with social media data. The compound score is computed by summing the valence scores of each word in the lexicon, adjusted according to the rules, and then normalized to be between -1 (most extreme negative) and +1 (most extreme positive). Some examples of VADER scoring results^13^ are shown in Table 3.

**Table 3:** Some examples of VADER scoring results

Examples of tweets and VADER scoring
VADER is VERY SMART, uber handsome, and FRIGGIN FUNNY!!!
{‘pos’: 0.706, ‘neu’: 0.294, ‘neg’: 0.0, ‘compound’: 0.9469}
Today only kinda sux! But I’ll get by, lol!
{ ‘pos’: 0.317, ‘neu’: 0.556, ‘neg’: 0.127, ‘compound’: 0.5249}
Make sure you :) or :D today!
{‘pos’: 0.706, ‘neu’: 0.294, ‘neg’: 0.0, ‘compound’: 0.8633}
VADER is not smart, handsome, nor funny.
{‘pos’: 0.0, ‘neu’: 0.354, ‘neg’: 0.646, ‘compound’: -0.7424}

We set a standardized threshold for classifying sentences as positive, neutral, or negative, as follows:

1 $$\begin{aligned} S_{f_i}= \left\{ \begin{array}{ccc} positive &{} v_{score} >=0.05, \\ negative &{} v_{score} <=-0.05, \\ neutral &{} otherwise, \end{array}\right. \end{aligned}$$

where $v_{score}$ is the compound score of the *i*-th tweet, $S_{f_i}$ is the final polarity of tweet. If the compound score $v_{score}$ is not less than 0.05, the sentence is considered to be positive. If the score is not greater than -0.05, its polarity is negative. Otherwise, the sentence polarity is neutral. Table 4 shows examples of tweets with positive and negative sentiment scores computed with VADER in this study.

**Table 4:** Examples of tweets with positive/negative sentiment

Tweets	Sentiment
Thanks to the vaccines, i was able to give my grandma a hug today for the first time in a long time.	Positive
Got my 2nd shot yesterday; my arm hurts a little more than after the 1st, but glad to be fully vaccinated.	Positive
I received the first vaccine. thank you and i am grateful.	Positive
Just received my second dose of happy dance to commence.	Positive
Its been a month since my dose number two and i am concerned that my shoulder might be permanently jacked up.	Negative
I am lost for words with reports that people in the eu are refusing the vaccine.	Negative
My second dose of of is due in 4 days and there is no stock or dates available. what do i do now?	Negative
11.5 hours later my arm hurts and the upper part is visibly swollen and i can feel a large lump.	Negative

### Topic modeling of tweets

**Fig. 4:**
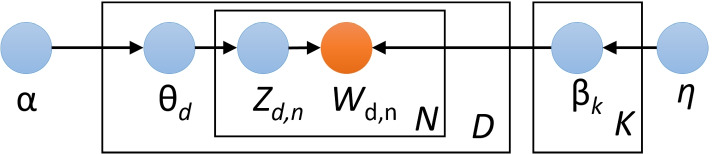
A plate notation explanation of LDA

Topic modeling is a method for the unsupervised classification of documents. Specifically, it’s the process of learning, recognizing, and extracting high-level semantic topics across a corpus of unstructured text even when people are unsure what they are looking for. It is a great way to get a bird’s-eye view of a large text collection. The most popular topic model is Latent Dirichlet Allocation (LDA) proposed by Blei et al. [3], LDA aims to find topics a document belongs to, based on its words. LDA is based on a Bayesian probabilistic model where each topic has a discrete probability distribution of words, and each document is composed of a mixture of topics. In LDA, the topic distribution is assumed to have a Dirichlet prior, giving a smooth topic distribution for each document. The probability for a corpus is modeled in Eq. 2, where the documents and words are assumed to be independent. We show the plate notation explanation of LDA in Figure 4 while the meaning of the notations is shown in Table 5.

2 $$\begin{aligned} \prod \limits _{d=1}^{N_d}P({w_1},\cdot \cdot \cdot ,{w_{N_d}}|\beta ,\alpha )= \prod \limits _{d=1}^{N_d}\int _{\theta _d}P({\theta _d}|\alpha )\left\{ \prod \limits _{n=1}^{N_d}\left( \underset{k}{\sum }{\theta _{dk},\beta _{kw_n}}\right) \right\} d{\theta _d} \end{aligned}$$

**Table 5:** Meaning of the notations

Symbol	Description
*K*	total number of topics
*D*	total number of documents
*N*	total number of words in a document
$\alpha ,\eta$	Dirichlet parameters
$\theta _d$	per-document topic proportions
$Z_{d,n}$	per-word topic assignment
$W_{d,n}$	observed word
$\beta _{k}$	topic, a distribution over the vocabulary

LDA assumes the following generative process for a corpus *D* consisting of *M* documents each of length $N_i$:

1. Generate ${\theta _i}\sim Dir({\alpha })$, where $i \in \left\{ 1,2,\cdot \cdot \cdot ,D\right\}$. $Dir({\alpha })$ is a Dirichlet distribution with symmetric parameter ${\alpha }$ where ${\alpha }$ is often sparse.
2. Generate ${\beta _k} \sim Dir ({\eta })$, where $k \in \left\{ 1,2,\cdot \cdot \cdot ,K\right\}$ and ${\beta }$ is typically sparse.
3. For the $n_{th}$ position in document *d*, where $n \in \left\{ 1,2,\cdot \cdot \cdot ,N_d\right\}$ and $d \in \left\{ 1,2,\cdot \cdot \cdot ,D\right\}$.
  - Choose a topic $z_{d,n}$ for that position which is generated from $z_{d,n} \sim Multinomial ({\theta _i}$)
  - Fill in that position with word $w_{d,n}$ which is generated from the word distribution of the topic picked in the previous step $w_{i,j} \sim Multinomial (\theta _{z_{d,n}})$

In this study, we employ LDA for topic modeling and discuss hot topics in positive and negative tweets separately. The number of topics is a crucial parameter in topic modeling. To make these topics human interpretable, we use the coherence score to determine the optimal number of topics. The coherence score in the following Eq. 3 helps to distinguish between human understandable topics and artifacts of statistical inference:

3 $$\begin{aligned} Coherence=\underset{i<j}{\sum }score(w_i,w_j). \end{aligned}$$

**Fig. 5:**
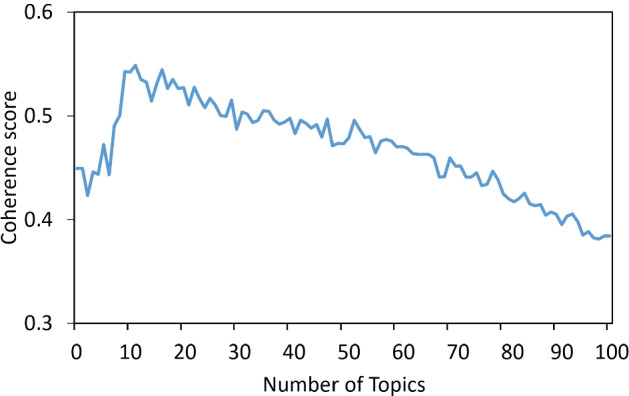
Coherence scores corresponding to the different number of topics

The coherence selects top *n* frequently occurring words in each topic, then aggregates all the pairwise scores of the top *n* words $w_i,\cdot \cdot \cdot ,w_n$ of the topic. Finally, we can get the total coherence score of the current number of topics. Figure 5 displays the coherence score of all tweets for the number of topics across two validation sets, and a fixed $\alpha = 0.01$ and $\beta = 0.1$. We set the range of the number of topics from 1 to 100. According to the results, the coherence score is highest when the number of topics is 11, so we determine the number of topics to be 11, and then perform LDA topic modeling on the tweets.

## Experimental results and analysis

We first look at the high-frequency vocabulary in the dataset, and then we extract prevalent words in tweets concerning user location and vaccine brands. After that, we use VADER to generate the sentiment polarity of each tweet, namely positive, negative and neutral, and then further analyze the attitudes of users in various countries to the seven vaccines. Finally, we use the LDA topic model to generate the topics of positive and negative tweets and examine the hot topics discussed in the tweets, respectively.

### Prevalent words by countries

**Fig. 6:**
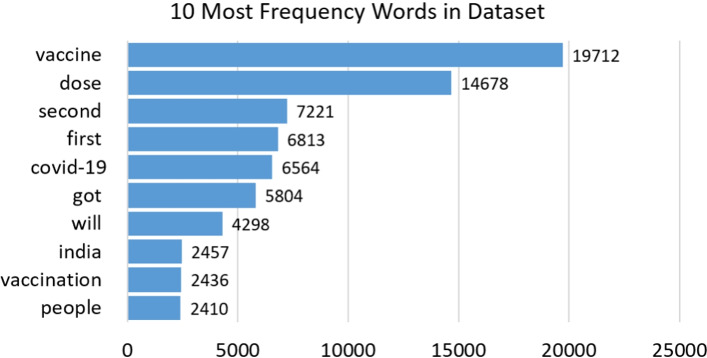
The 10 most high-frequency words in the dataset

After removing the stopwords and meaningless words, we first count the high-frequency vocabularies in the dataset, as shown in Figure 6. Then, we separately count the popular words in the discussions of the COVID-19 vaccine in different countries.

**Fig. 7:**
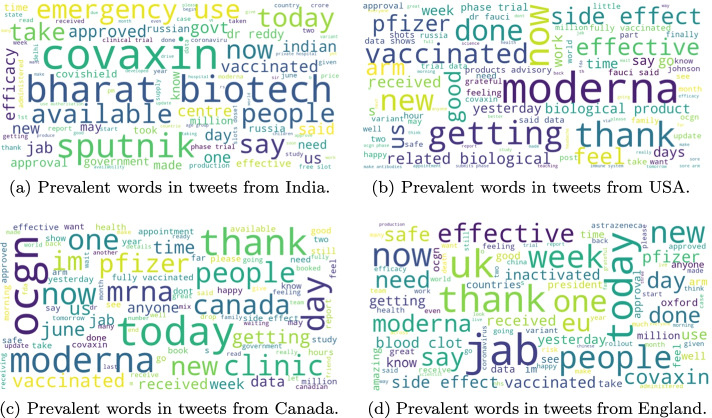
Prevalent words in tweets from four countries in the dataset

We extract prevalent words from tweets in India, USA, Canada, England and then use the word cloud to visualize them, as shown in Figure 7. According to Table 2, we learn that India has approved 4 of the 7 vaccines, USA has approved 2 of the 7 vaccines, Canada and England have approved 3 of the 7 vaccines. Figure 7a clearly shows that Indian people pay more attention to Bharat biotech (Covaxin) and Sputnik than Moderna and Oxford/AstraZeneca. In USA, Canada and England, Moderna and Pfizer are the most mentioned vaccines by users. The word “thank” is clearly visible in the word cloud, showing a positive attitude, as shown in Figure 7b, c, and d.

### Prevalent emotional words by vaccines

In this section, we pay attention to the high-frequency emotional vocabularies related to vaccines and gain a general understanding of people’s attitudes toward different vaccines. We employ the VADER dictionary to filter the emotional words in the tweets, select the top 30 high-frequency emotional words for each vaccine, and then separate the words by polarity. The results are shown in Tables 6 and 7, and we can see that the number of positive words is much higher than the number of negative words, such as “thank”, “approved”, “effective”, “safety”, “hope”. These words represent a positive attitude towards vaccines, trusting vaccines can protect us from infection. We did not list neutral words because only two neutral words were mentioned in all vaccines’ top 30 high-frequency emotional words.

**Table 6:** High-frequency positive words of different vaccines in tweets

Vaccine	Positive Vocabulary
Pfizer/BioNTech	effective like thank good thanks approved great feeling want safe heart grateful well better happy protect please best ready hope approves share
Sinopharm	well boost want special great ready approved effective good free approval thank like approves safe help please thanks better positive support number feeling gift best
Sinovac	approved validated approves reaches best feeling launched like better effective thank approval good safe thanks well want validates number specialplease successfully boost
Oxford/AstraZeneca	proud feeling effective safety good safe pleased happy great thank like hope delighted approved thanks well grateful want fine amazing please
Moderna	feeling ready thanks grateful great better like best thank safe approval hope please good effective happy number free want approved well excited super help
Covaxin	safe best dear well want help trust effectively positive good approval better like thanks effective great immune proud please approved thank free hope top
Sputnik V(Gamaleya)	supreme number approved well help ready thank thanks free allow best trust good effective like want approval great top launched approves please agreed

**Table 7:** High-frequency negative words of different vaccines in tweets

Vaccine	Negative Vocabulary
Pfizer/BioNTech	no emergency risk warning death refused
Sinopharm	emergency low no missed
Sinovac	no low emergency death died
Oxford/AstraZeneca	no stop risk suspend sore rejected ill
Moderna	no ill sore emergency pain
Covaxin	severe strain emergency no shortage
Sputnik V(Gamaleya)	no emergency demand death fight

### Sentiment analysis of tweets

**Fig. 8:**
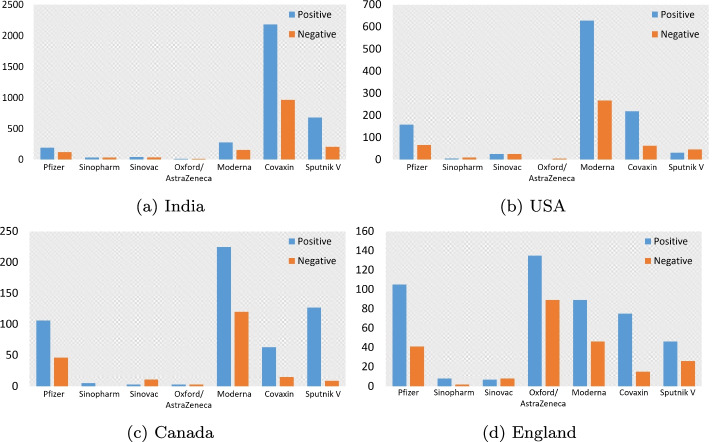
Sentiment analysis of tweets for different vaccine brands in India, USA, Canada and England

The sentiment polarity of each tweet is generated using the VADER tool as described previously. Figure 8 presents the overall emotional distribution of tweets across the four countries to the seven vaccines over the study period. Obviously, it can be seen that the number of positive tweets is greater than that of negative tweets, regardless of the brand, which shows that the majority of Twitter users maintain a positive attitude towards the vaccines. According to Table 6, most of the positive tweets focused on the following aspects, such as believing that vaccines can provide effective protection, expecting that the vaccine will be approved and promoted as soon as possible, thanking the injection of the vaccine. In contrast, negative tweets are mostly related to vaccine shortages, side effects after vaccination, and reports of deaths due to vaccination.

**Table 8:** The most discussed topics in positive tweets about the COVID-19 vaccine on Twitter during the study period

Topic 1	Topic 2	Topic 3	Topic 4	Topic 5
vaccine	feel	second	go	get
say	today	shoot	people	be
well	day	shot	make	still
able	good	thank	see	happy
friend	week	amp	safe	stay
come	time	arm	vaccinate	last
many	great	do	may	vaccinated
free	yesterday	vaccination	work	look
soon	thing	hit	let	tell
start	back	ready	help	lot

**Table 9:** The most discussed topics in negative tweets about the COVID-19 vaccine on Twitter during the study period

Topic 1	Topic 2	Topic 3	Topic 4	Topic 5
vaccine	arm	be	get	take
first	dose	shot	week	people
day	sore	go	ill	know
feel	second	amp	report	stay
still	shoot	make	find	home
pain	yesterday	may	kill	vaccination
fever	have	update	hear	much
little	time	come	state	good
tired	body	think	severe	month
die	tell	vaccinate	expect	will

### Topic modeling of tweets

As mentioned in Section 4.2, we use the coherence score to determine the optimal number of topics for topic modeling is 11. In this section, we use LDA to generate the topics of the tweets to understand which aspects users concern about in the positive and negative tweets, respectively. We count the number of tweets corresponding to different topics in positive tweets and negative tweets separately. According to the popularity, the top 5 topics discussed in positive and negative tweets are listed in Tables 8 and 9, where the most contributing words related to the topic are shown below the topic in the Tables. We get the consistent conclusions as in Sections 5.2 and 5.3. In the positive tweets, people were grateful for being vaccinated in anticipation of returning to normal life; in negative tweets, most of them complain about side effects after vaccination, such as fever, sore arm, etc.

## Conclusion

This study conducted a comprehensive analysis of COVID-19 vaccine-related tweets collected from Twitter between December 12, 2020 and July 2, 2021. A total of 75,665 COVID-19 vaccine related tweets were used for this study. According to statistics based on the location of tweet users, these tweets were mainly posted by users in four countries: India, USA, Canada, and England. We first performed an overall analysis on the whole dataset and then a specific analysis for the four countries. The sentiment analysis results showed that the overall sentiment polarity is positive, and the number of positive tweets is approximate twice as much as the number of negative tweets. When we drilled into country-level, it was found that the sentiment polarity scores of each country for the approved vaccines were consistent with the overall sentiment polarity scores. But when it came to other vaccine brands, the number of negative tweets for some vaccines is higher than positive tweets, such as Sputnik V in USA and Sinovac in Canada and England. In the positive tweets, people expressed their gratitude for being able to be vaccinated. They hope that with the help of the vaccination, the pandemic can be controlled as soon as possible and normal life can be resumed. What is more, we found that people mostly complained about side effects after vaccination in the negative tweets, such as fever, sore arm, etc.

In summary, this paper presented a case study of popular topics and sentiment analysis of tweets related to the COVID-19 vaccines. In the future, more interesting topics can be explored based on the current study. For example, conducting individual-level topic and sentiment analysis can identify people who may be affected by negative emotions, which can help local governments or agencies to understand their clients with more explainable information, and consider the necessary intervention.

## Footnotes

1 https://www.who.int/health-topics/coronavirus#tab=tab_3

2 https://www.who.int/health-topics/coronavirus#tab=tab_1

3 https://covid19.who.int/

4 https://covid19.trackvaccines.org/

5 https://www.who.int/news/item/31-12-2020-who-issues-its-first-emergency-use-validation-for-a-covid-19-vaccine-and-emphasizes-need-for-equitable-global-access

6 https://ourworldindata.org/covid-vaccinations

7 https://www.who.int/health-topics/infodemic

8 https://www.kaggle.com/gpreda/all-covid19-vaccines-tweets

9 https://covid19.trackvaccines.org/vaccines/approved/#vaccine-list

10 https://www.bbc.com/news/world-asia-india-56912977

11 https://covid19.trackvaccines.org/trials-vaccines-by-country/

12 https://ourworldindata.org/covid-vaccinations

13 https://github.com/cjhutto/vaderSentiment
